# On the Different Methods of Treating Intermittent Fever, with the Results of Treatment in Sixty-Nine Cases

**Published:** 1857-01

**Authors:** Austin W. Nichols

**Affiliations:** Buffalo


ART. II.— *On the Different Methods of Treating Intermittent Fever, with the Results of Treatment in Sixty-nine Cases.* By AUSTIN W. NICHOLS, M. D., Buffalo.

At a certain period, that plan of treating intermittent fever which was universally employed by the profession, was the preparation of the system for the introduction of quinia, by the previous use of emetics or cathartics, or both. By the administration of these remedies, the intention was to remove the contents of the stomach and intestines, especially to cleanse the former organ and to purge away the ever present bile, so that there would be no hindrance to the immediate absorption of the quinia whenever it should be introduced.

Of late years, a change has taken place in the opinion of many practitioners, more generally amongst those of our northern states, regarding the utility of this preparatory course of treatment. The necessity for it has been disavowed; and quinia has been given at once and in very large doses, without any of the supposed ill effects which those who believed in the preparation of the patient, had attributed to its immediate employment.

At an early date, Dr. Flint, of this city, advocated the more recent plan of treating this disease; and a very interesting article upon the subject, from his pen, will be found in the American Journal of Medical Sciences, October, 1841. The results of the treatment of this disease from the immediate introduction of the quinia, and in extremely large doses in some cases, are there given, in connection with many other points of importance.

In our middle and southern states, however, the old method of treating intermittent fever, is still generally in use. Every year, most of the patients there affected, are still placed on the use of the emetic and cathartic class of medicines, and quinia only given after their operation. But it has been long known, that many relapses occur, that the cures are not permanent: and this has been attributed to the impurity of the quinia, to the peculiar form of the fever, and to a tendency in the disease to relapse at that particular season of the year. Doubtless, there may be some truth in each of these suppositions. May not a tendency to relapse, however, be increased (if not in reality produced) by the plan of treatment pursued?

It appears rational to presume, that the sooner the paroxysms are arrested, the less will the system have been debilitated by the cause of intermittent fever. It may be affirmed, with safety, that the administration of emetics or cathartics, does not tend to improve the tone or vigor of the body; these remedies debilitate rather than strengthen or invigorate. Can we, therefore, not merely arrest the paroxysms, but protect the patient against so great a tendency to relapse, by the immediate introduction of the quinia, and avoid any ill effects?

The following analysis of 69 cases of intermittent fever, is submitted in order to demonstrate, as far as these few cases are capable of doing, the results of the different methods of treatment pursued:

Of the 69 cases, the duration of 63 was $394\frac{11}{12}$ days, the duration of 6 cases being unknown: average, about $6\frac{1}{3}$ days.

In the 63 cases, the number of paroxysms was 225: average $3\frac{4}{7}$.

In 64 cases, in which the relapse was noted, there were 23 cases of relapse: average about 1 in 3.

Of the 69 cases, 22 had had one or more previous attacks; and 47 were recent attacks.

Of the 22 cases, 20 cases lasted 138 days, and there occurred 11 relapses; 2 cases being unknown: average duration of 20 cases of prior attacks, $6\frac{9}{10}$ days: average frequency of relapses, about $\frac{1}{2}$.

Of the 47 cases, the duration and number of relapses, is noted in 43 cases and 44 cases respectively; in the 43 cases, the duration was $256\frac{11}{12}$ days: average duration of recent cases, about 6 days. In the 44, the cases of relapse numbered 12: average, 1 in 4. Total of these two classes, 22 prior attacks and 47 recent attacks, duration $6\frac{9}{10}$ and 6 days respectively; relapses in $\frac{1}{2}$ of the 22, and $\frac{1}{4}$ of the 47 cases.

The 69 cases are divided into two classes, class first containing 46 cases, or those treated in a section of country where intermittent fever prevails to a great extent every year; and class second, of 23 cases treated in a section of country where this disease prevails to a very small extent every year, or in a region not malarious.

### CLASS FIRST.

46 cases, all being private patients: 30 males and 16 females; 17 had no preparatory treatment, and 29 had a preparatory treatment consisting of ipecacuanha and calomel, calomel and rhubard, or jalap combined.

| *Types of the* 17 *Cases.* | *Types of the* 29 *Cases.* |
|---|---|
| 1, no type determined; | |
| 2, quotidian; | 2, quotidian; |
| 13, tertian; and | 27, tertian; and |
| 1, quartan. | 1, every 7th day. |

*No. of Paroxysms in each Type.*

2 cases of quotidian:
1 had 6 paroxysms,
1 had 7 "

13 cases of tertian:

2 had 2 paroxysms,

3 " 3 "

1 " 4 "

3 " 5 "

2 " 6 "

2 were unknown.

1 quartan case had 4 paroxysms.

*No. of Relapses in each Type.*

1 in the quotidian;
3 in the tertian.

*Duration of Cases in each Type.*

| | | |
|---|---|---|
| 1 undetermined type, | | 1 day; |
| 2 cases of quotidian, | | 13 days; |
| 11 " " tertian, | | 77 " |
| (2 " " " | | unknown;) |
| 2 " " quartan, | | 4 days. |
| 15 cases: duration, | | 95 days: |
| average of 15 cases, | | $6\frac{1}{3}$ days. |

*No. of Paroxysms in each Type.*

1 case of quotidian had 14 paroxysms.

27 cases of tertian:

6 had 2 paroxysms,

10 " 3 "

6 " 4 "

2 " 5 "

1 " 6 "

1 " 7 " and

1 "10 "

1 " every 7th day case " had 7 paroxysms.

*No. of Relapses in each Type.*

1 in the quotidian;
12 in the tertian;
1 in "every 7th day."

*Duration of Cases in each Type.*

| | |
|---|---|
| 1 case of quotidian, | 14 days; |
| 27 cases tertian, | 171 " |
| 1 case "every 7th day," | 43 " |
| 29 cases: duration, | 228 days: |
| average of 29 cases, | $7\frac{25}{29}$ days. |

But, to the duration of the 29 cases, 406 hours, or $16\frac{11}{12}$ days, will have to be added: this being the time consumed in the preparatory treatment, previous to the exhibition of any quinia:

| | |
|---|---|
| The quinia treatment of 29 cases, - - - | 228 days. |
| The preparatory " " " " - - - - | $16\frac{11}{12}$ " |
| The total treatment, duration of, - - - | $244\frac{11}{12}$ days. |
| Average duration of 29 cases, - - - - | $8\frac{1}{3}$ " |
| " " " 15 " - - - - - | $6\frac{1}{3}$ " |

We will now compare the cases in regard to the number of paroxysms, the total duration, and the number of relapses, regarding the cases as still farther divisible into cases of recent and prior attacks; for prior attacks, we have found, to influence slightly the duration and in a marked degree the number of relapsing cases.

| Cases, No. of. | | No. of Paroxysms. | Average of. | Duration. | Average of. |
|---|---|---|---|---|---|
| n.p.t. 15 | 10 recent, | Total, 61; | $4\frac{1}{15}$; | 56 days; | $5\frac{3}{5}$ days. |
| | 5 prior, | | | 39 " | $7\frac{4}{5}$ " |
| p.t. 29 | 22 recent, | Total, 120; | $4\frac{4}{29}$; | $175\frac{11}{12}$ days; | 8 days. |
| | 7 prior, | | | 69 " | $9\frac{6}{7}$ " |

| Cases, No. of. | No. of Relapses. | Averages. |
|---|---|---|
| 17 no prep. treat. | 4 cases. | 1 in every $4\frac{1}{4}$. |
| 29 with prep. treat. | 14 " | 1 " " $2\frac{1}{14}$. |

In order to arrive at greater accuracy, we will compare the cases of tertian type by themselves; discarding from the 17 cases treated with quinia alone, the single case of undetermined type, the two cases of quotidian, and the case of quartan; and from the 29 cases treated by a preparatory course of medicine, the case of quotidian and the case occurring every 7th day.

| No. of Cases. | Quinia Treat't. | Average of | Total Treat. | Average of. |
|---|---|---|---|---|
| n. p. t. 11 | 77 days. | 7 days. | 77 days. | 7 days. |
| p. t. 27 | 171 " | $6\frac{3}{4}$ " | 184 " | $6\frac{22}{27}$ " |

| No. of Cases. | No. of Paroxysms. | Averages. | Relapses. | Averages. |
|---|---|---|---|---|
| n. p. t. 13 | (11 cases,) 44, | 4, | 3 cases, | 1 in $4\frac{1}{3}$. |
| p. t. 29 | 99, | $3\frac{2}{3}$, | 12 " | 1 in $2\frac{1}{3}$. |

"n. p. t.", no preparatory treatment; "p. t.", preparatory treatment.

### CLASS SECOND.

23 cases: all but two hospital cases; 22 males and 1 female; all having had no preparatory treatment. 21 of these cases occurred in the "Male General Ward" of the hospital, of which Dr. Flint has charge.

| *Types of the 23 Cases.* | *No. of Paroxysms in each Type.* |
|---|---|
| 1, no type determined, | 1, undetermined type, |
| 12, quotidian, | 29, in 11 cases of quotidian, |
| 3, double quotidian, | 16, " 3 " double " |
| 7, tertian. | 9, " 4 " tertian. |

| *Duration in each Type.* | | | *No. of Cases of Relapses in each Type.* |
|---|---|---|---|
| 1 day, undetermined type, | | | |
| 29 days, quotidian; | 11 cases, | | 3 cases, in 11 quotidian, |
| 8 " double " | 3 " | | 1 case, in 3 double " |
| 17 " tertian, | 4 " | | 1 " " 4 tertian. |

| No. of Cases. | No. of Paroxysms. | Average No. |
|---|---|---|
| 19 | 55 | $2\frac{17}{19}$. |

| No. of Cases. | Duration. | Average Do. | Cases of Relapse. | Averages. |
|---|---|---|---|---|
| 19 | 55 days. | $2\frac{17}{19}$ days. | 5 of 18 cases. | 1 in $3\frac{3}{5}$. |

Of these 23 cases, 10 had prior attacks and 13 were recently attacked; of 9 cases of "prior attack," the duration was 30 days, and there were 3 cases of relapse. Of the 13 "recent cases," in 10 the duration was 25 days, in 3 unknown, and there were 2 cases of relapse.

| No. of Cases. | Duration of. | Average of. | Cases of Relapse. |
|---|---|---|---|
| 10 recent, | 25 days, | $2\frac{1}{2}$ days, | 2 |
| 9 prior, | 30 " | $3\frac{1}{3}$ " | 3 |

Comparing the cases in each of the two classes, having no preparatory treatment, the result is vastly in favor of that region which is not malarious.

| Class. | No. of Cases, | Aver. Duration. | Av. No. Parox. | Aver. Cases of Relapse. |
|---|---|---|---|---|
| 1 | 15 | $6\frac{1}{3}$ days, | $4\frac{1}{15}$ | 1 in $4\frac{1}{4}$ (17 cases.) |
| 11 | 19 | $2\frac{17}{19}$ " | $2\frac{17}{19}$ | 1 in $3\frac{3}{5}$ (18 cases.) |

During the past summer and fall, five cases of intermittent fever, of a rare type, have been treated in the "male general ward" of the hospital; these were of the *double quotidian* type: a paroxysm commencing with rigor about sunrise, fever and perspiration succeeding, and terminating about noon; another paroxysm commencing with rigor, about 3 or 4, or as late as 6, P. M., fever and perspiration following, and terminating near midnight. On the following day, the same circumstances taking place, two distinct paroxysms coming on at certain periods of the day. Three of these cases are included in class 11: two occurred before these observations were commenced. They obeyed the same law which has been found to govern cases of other types of this fever, namely, that the longer the duration of the disease or the more frequent the attack, the more difficult will be the cure and the greater the tendency to relapse.

Of the five cases, two had continued for some time before they applied for treatment; both of these cases had frequent relapses, and both required large doses of quinia, the highest dose being grs. x, three times daily. Three cases had been attacked but two or three days; one of these had a single relapse, the greatest number of grains of quinia being v, three times daily.

At times, the system of a patient seems to be unaffected by very large doses of quinia continued for several days; in class ii, in a case of quotidian, the paroxysm recurring every afternoon, 192 grains of quinia were given before any effect was noticeable; the greatest number of grains administered in a single day, "xxxvi"; in a single dose, "xii;" this amount of quinia produced none of its usual effects upon the nervous system, such as ringing in the ears, &c. But the paroxysms were arrested. This patient had a vesicular eruption on the lips, previous to the period at which the paroxysms were arrested.

Of the 69 cases, 1 had just recovered from typhoid fever, of a mild form; 1 was affected with emphysema; 3 had considerable enlargement of the spleen. 34 cases presented a highly anæmical appearance, and 35 cases appeared to be healthy, during the intermission between the paroxysms. The vesicular eruption on the lips was present in 11 of 69 cases; in 3 of the 40 cases with no preparatory treatment; in 8 of the 29 cases with preparatory treatment.

The quinia employed in Class 1, was from the manufactory of Rosengarton & Sons; highest number of grains given in a single dose, viii.

The quinia employed in Class ii, was from the manufactory of Powers & Weightman; highest number of grains given in a single dose, xii.

### *Summary.*

From the foregoing analysis, it will be seen that in cases of first attack, the duration is somewhat less, and the number of cases relapsing about one half that in cases having had one or more prior attack. In a section of country where this fever is prevalent to a great degree every year, or in a malarious region, the duration is nearly three times that in a country not malarious, and the relapsing cases occur as frequently, even under the same plan of treatment. It will be found, that in a malarious region the treatment of patients by quinia alone, not only diminishes the number of paroxysms and abridges the duration of the disease, but that fewer relapsing cases occur, than where a preparatory course of treatment had been adopted. Even in the analysis of the 40 tertian cases, although the duration and the number of paroxysms are nearly the same under the two different methods of treatment employed, yet the cases of relapse are found to be *nearly twice* as numerous where the preparatory plan was adopted.

